# Supplementary material for: Association between oral microbiome and seven types of cancers in East Asian population: a two-sample Mendelian randomization analysis
Source: Front Mol Biosci. 2023 Nov 21;10:1327893. doi: 10.3389/fmolb.2023.1327893 (PMC10702768; doi:10.3389/fmolb.2023.1327893)
Supplement: Supplementary file 1 [file DataSheet1.ZIP › Supplementary Materials/MR plots of saliva/saliva═╝/colorectal cancer/pheno.2027_to_colorectal cancer_leave_one_out.pdf]

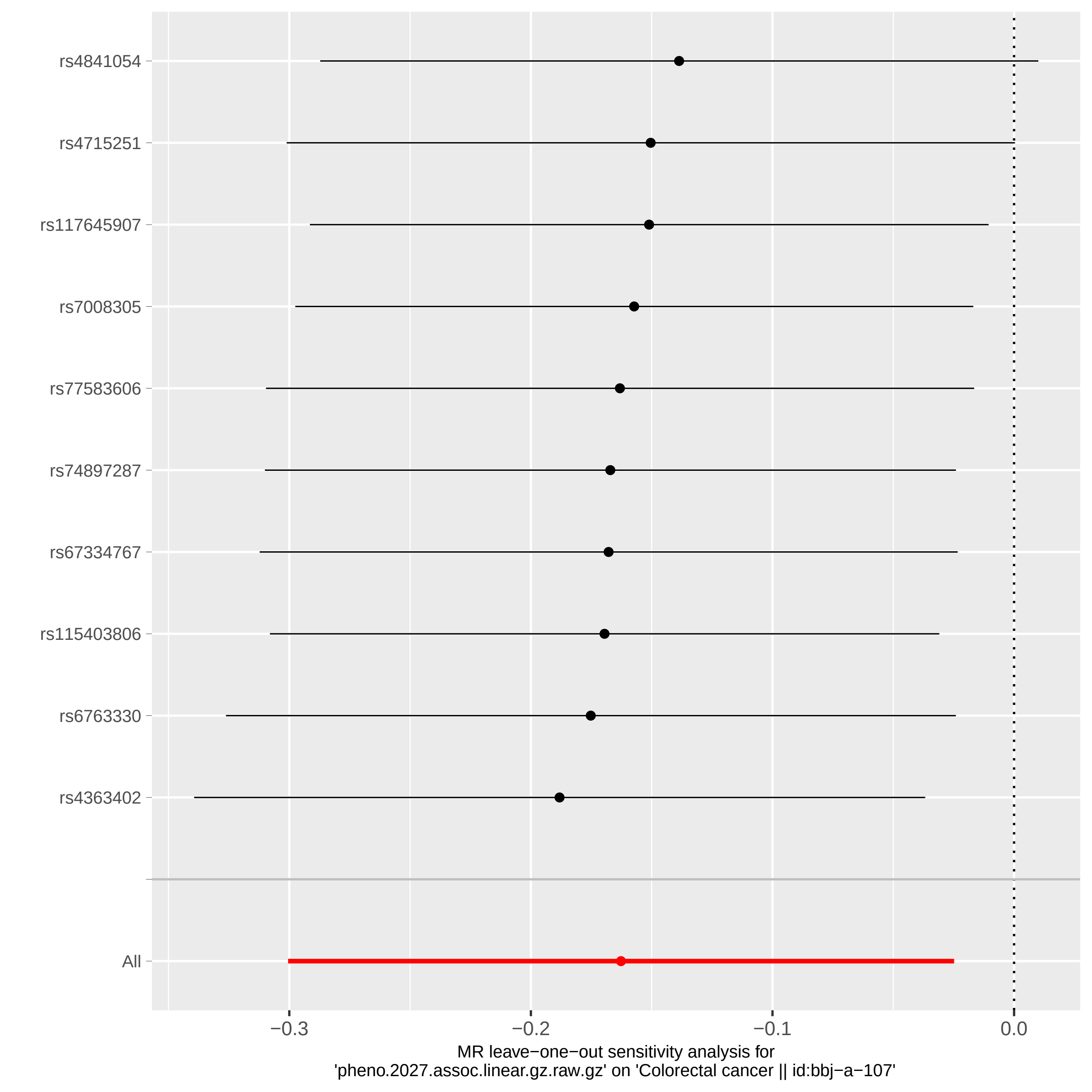
rs4841054
rs4715251
rs117645907
rs7008305
rs77583606
rs74897287
rs67334767
rs115403806
rs6763330
rs4363402
All
−0.3
−0.2
−0.1
0.0
MR leave−one−out sensitivity analysis for
'pheno.2027.assoc.linear.gz.raw.gz' on 'Colorectal cancer || id:bbj−a−107'
